# 6,7-Dimeth­oxy-1,4-anthraquinone

**DOI:** 10.1107/S1600536808026500

**Published:** 2008-08-23

**Authors:** Chitoshi Kitamura, Naoki Akamatsu, Akio Yoneda, Takeshi Kawase

**Affiliations:** a Department of Materials Science and Chemistry, Graduate School of Engineering, University of Hyogo, 2167 Shosha, Himeji, Hyogo 671-2280, Japan

## Abstract

The mol­ecule of the title compound, C_16_H_12_O_4_, is almost planar; the two meth­oxy groups are slightly out of the plane of the anthraquinone ring system, with C—C—O—C torsion angles of −6.25 (19) and −10.22 (19)°. In the crystal structure, the mol­ecules adopt a herringbone arrangement and form face-to-face slipped anti­parallel π–π stacking inter­actions along the *b* axis, with an inter­planar distance of 3.278 (2) Å.

## Related literature

For the synthesis of 1,4-anthraquinone, see: McOmie & Perry (1973 ▶). For related structures, see: Kitamura *et al.* (2006 ▶).
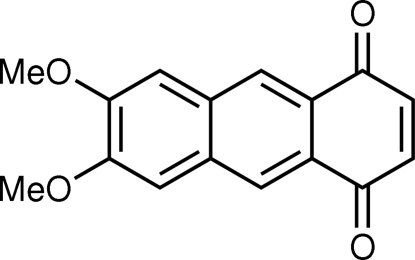


## Experimental

### 

#### Crystal data

C_16_H_12_O_4_
*M* *_r_* = 268.26
Monoclinic, 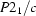

*a* = 7.478 (3) Å
*b* = 7.492 (3) Å
*c* = 22.949 (9) Å
β = 106.646 (10)°
*V* = 1231.8 (8) Å^3^
*Z* = 4
Mo *K*α radiation
μ = 0.10 mm^−1^
*T* = 223 K
0.5 × 0.1 × 0.1 mm

#### Data collection

Rigaku/MSC Mercury CCD area-detector diffractometer
Absorption correction: numerical (**NUMABS**; Higashi, 1999 ▶) *T*_min_ = 0.974, *T*_max_ = 0.991
5255 measured reflections
2705 independent reflections
2177 reflections with *I* > 2σ(*I*)
*R*_int_ = 0.018

#### Refinement

*R*[*F* ^2^ > 2σ(*F* ^2^)] = 0.049
*wR*(*F* ^2^) = 0.147
*S* = 1.09
2705 reflections
181 parameters
H-atom parameters constrained
Δρ_max_ = 0.28 e Å^−3^
Δρ_min_ = −0.17 e Å^−3^

### 

Data collection: *CrystalClear* (Rigaku/MSC, 2001 ▶); cell refinement: *CrystalClear*; data reduction: *WinGX* (Farrugia, 1999 ▶); program(s) used to solve structure: *SIR2004* (Burla *et al.*, 2005 ▶); program(s) used to refine structure: *SHELXL97* (Sheldrick, 2008 ▶); molecular graphics: *ORTEP-3 for Windows* (Farrugia, 1997 ▶); software used to prepare material for publication: *WinGX*.

## Supplementary Material

Crystal structure: contains datablocks global, I. DOI: 10.1107/S1600536808026500/is2326sup1.cif

Structure factors: contains datablocks I. DOI: 10.1107/S1600536808026500/is2326Isup2.hkl

Additional supplementary materials:  crystallographic information; 3D view; checkCIF report

## Figures and Tables

**Fig. 1.**
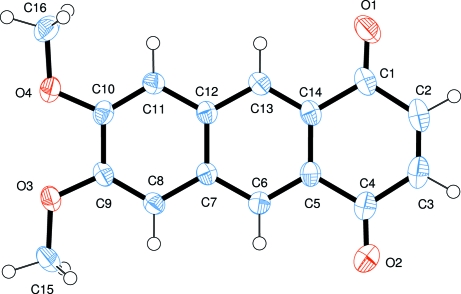
The molecular structure of (I), showing 50% probability displacement ellipsoids for non-H atoms.

**Fig. 2.**
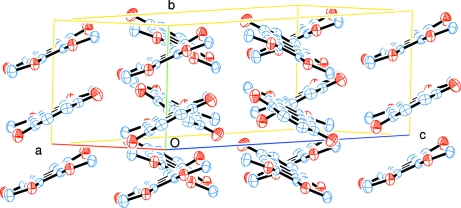
The packing diagram of (I). Hydrogen atoms are omitted for clarity.

## supplementary crystallographic information

### Comment

1,4-Anthraquinone has a weak dipole moment along the molecular long axis, whose value was estimated to be 2.54 debye by a B3LYP/6–31G(*d*) DFT calculation (Kitamura *et al.*, 2006). The crystal structure exhibited a herring-bone packing with face-to-face slipped π-overlap along the stacking direction. In addition, the molecules were antiparallel with respect to one another. These charasteristics inspired us to study other 1,4-anthraquinone derivatives. The title compound (I), which was first prepared by McOmie & Perry (1973), has strong electron-donating methoxy groups, and therefore, has a larger dipole moment, compared with 1,4-anthraquinone, which was estimated to be 3.91 debye. This property should affect the intermolecular interactions in the crystal.

The molecular structure of (I) is shown in Fig. 1. The molecule is an almost coplanar conformation. The displacements of atoms O1, O2, O3, O4, C15, and C16 relative to the plane of the anthracene backbone are 0.159 (2), -0.004 (2), -0.039 (2), -0.003 (2), 0.168 (2), and -0.145 (2) Å, respectively. The torsion angles of the two methoxy groups are -6.25 (19)° for C11—C10—O4—C16 and -10.22 (19)° for C8—C9—O3—C15, indicating that the C_methyl_—O bonds are directed along the molecular short axis. As shown in Fig. 2, the molecules adopt a herring-bone arrangement and form face-to-face slipped antiparallel π-π stacking along the direction of the *b* axis. The interplanar distance is 3.278 (2) Å, whose value is shorter than that (3.423 Å) of 1,4-anthraquinone (Kitamura *et al.*, 2006) and is indicating the existence of strong intermolecular interactions.

### Experimental

The title compound was prepared according to the modified method described by McOmie & Perry (1973). A mixture of 4,5-bis(bromomethyl)-1,2-dimethoxybenezene (340 mg, 1.05 mmol), 1,4-benzoquinone (571 mg, 5.28 mmol) and sodium iodide (797 mg, 5.31 mmol) in DMF (6 ml) was heated at 110 °C for 16 h. After the reaction mixture was cooled to room temperature, the mixture was decolorized by addition of aqueous 5% Na_2_SO_3_ solution. The resulting yellow precipitate was filtered off, washed with acetone, and dried under vacuum to give the crude product of (I) (74 mg, 26%). Yellow single crystals suitable for X-ray analysis were obtained by standing of a hot DMF solution of the crude product at room temperature.

### Refinement

All the H atoms were positioned geometrically (C_aromatic_—H = 0.94 and C_methyl_—H = 0.97 Å) and refined using a riding model, with *U*_iso_(H) = 1.2*U*_eq_(C) or 1.5*U*_eq_(methyl C).

### Crystal data

**Table:**

C_16_H_12_O_4_	*F*_000_ = 560
*M**_r_* = 268.26	*D*_x_ = 1.446 Mg m^−^^3^
Monoclinic, *P*2_1_/*c*	Melting point: 547 K
Hall symbol: -P 2ybc	Mo *K*α radiation λ = 0.71073 Å
*a* = 7.478 (3) Å	Cell parameters from 3335 reflections
*b* = 7.492 (3) Å	θ = 3.2–27.5º
*c* = 22.949 (9) Å	µ = 0.10 mm^−^^1^
β = 106.646 (10)º	*T* = 223 K
*V* = 1231.8 (8) Å^3^	Prism, yellow
*Z* = 4	0.5 × 0.1 × 0.1 mm

### Data collection

**Table:**

Rigaku/MSC Mercury CCD area-detector diffractometer	2705 independent reflections
Radiation source: rotating-anode X-ray tube	2177 reflections with *I* > 2σ(*I*)
Monochromator: graphite	*R*_int_ = 0.018
Detector resolution: 14.7059 pixels mm^-1^	θ_max_ = 27.5º
*T* = 223 K	θ_min_ = 3.3º
φ and ω scans	*h* = −9→9
Absorption correction: numerical(*NUMABS*; Higashi, 1999)	*k* = −9→9
*T*_min_ = 0.974, *T*_max_ = 0.991	*l* = −29→9
5255 measured reflections	

### Refinement

**Table:**

Refinement on *F*^2^	Hydrogen site location: inferred from neighbouring sites
Least-squares matrix: full	H-atom parameters constrained
*R*[*F*^2^ > 2σ(*F*^2^)] = 0.049	*w* = 1/[σ^2^(*F*_o_^2^) + (0.089*P*)^2^ + 0.0212*P*] where *P* = (*F*_o_^2^ + 2*F*_c_^2^)/3
*wR*(*F*^2^) = 0.147	(Δ/σ)_max_ < 0.001
*S* = 1.09	Δρ_max_ = 0.28 e Å^−^^3^
2705 reflections	Δρ_min_ = −0.17 e Å^−^^3^
181 parameters	Extinction correction: none
Primary atom site location: structure-invariant direct methods	

### Special details

**Table:**

Geometry. All e.s.d.'s (except the e.s.d. in the dihedral angle between two l.s. planes) are estimated using the full covariance matrix. The cell e.s.d.'s are taken into account individually in the estimation of e.s.d.'s in distances, angles and torsion angles; correlations between e.s.d.'s in cell parameters are only used when they are defined by crystal symmetry. An approximate (isotropic) treatment of cell e.s.d.'s is used for estimating e.s.d.'s involving l.s. planes.
Refinement. Refinement of *F*^2^ against ALL reflections. The weighted *R*-factor *wR* and goodness of fit *S* are based on *F*^2^, conventional *R*-factors *R* are based on *F*, with *F* set to zero for negative *F*^2^. The threshold expression of *F*^2^ > σ(*F*^2^) is used only for calculating *R*-factors(gt) *etc*. and is not relevant to the choice of reflections for refinement. *R*-factors based on *F*^2^ are statistically about twice as large as those based on *F*, and *R*- factors based on ALL data will be even larger.

### Fractional atomic coordinates and isotropic or equivalent isotropic displacement parameters (Å^2^)

**Table:**

	*x*	*y*	*z*	*U*_iso_*/*U*_eq_	
C1	0.2346 (2)	0.81897 (17)	0.17745 (6)	0.0348 (3)	
C2	0.4192 (2)	0.7551 (2)	0.21515 (7)	0.0439 (4)	
H2	0.4546	0.7773	0.2572	0.053*	
C3	0.5361 (2)	0.66760 (19)	0.19142 (7)	0.0429 (4)	
H3	0.6498	0.6269	0.2176	0.052*	
C4	0.4950 (2)	0.63168 (17)	0.12589 (6)	0.0362 (3)	
C5	0.30905 (19)	0.68684 (15)	0.08623 (6)	0.0286 (3)	
C6	0.26003 (18)	0.65203 (15)	0.02483 (6)	0.0280 (3)	
H6	0.3465	0.596	0.0081	0.034*	
C7	0.08283 (18)	0.69868 (15)	−0.01340 (6)	0.0254 (3)	
C8	0.03163 (18)	0.66243 (15)	−0.07661 (6)	0.0273 (3)	
H8	0.1177	0.6074	−0.0937	0.033*	
C9	−0.14174 (19)	0.70671 (16)	−0.11289 (6)	0.0285 (3)	
C10	−0.27351 (18)	0.79192 (15)	−0.08715 (6)	0.0289 (3)	
C11	−0.22806 (19)	0.82638 (16)	−0.02631 (6)	0.0285 (3)	
H11	−0.3163	0.88	−0.0098	0.034*	
C12	−0.04838 (18)	0.78199 (15)	0.01235 (6)	0.0263 (3)	
C13	0.00577 (19)	0.81899 (16)	0.07530 (6)	0.0292 (3)	
H13	−0.0794	0.8749	0.0926	0.035*	
C14	0.18036 (19)	0.77496 (16)	0.11181 (6)	0.0289 (3)	
C15	−0.0736 (2)	0.6124 (2)	−0.20281 (7)	0.0449 (4)	
H15A	−0.1353	0.593	−0.2457	0.067*	
H15B	−0.0186	0.5013	−0.1843	0.067*	
H15C	0.0235	0.7014	−0.1984	0.067*	
C16	−0.5822 (2)	0.9018 (2)	−0.10571 (7)	0.0413 (4)	
H16A	−0.6925	0.9256	−0.1393	0.062*	
H16B	−0.5399	1.0117	−0.0837	0.062*	
H16C	−0.6123	0.8153	−0.0785	0.062*	
O1	0.13258 (17)	0.90386 (15)	0.20045 (5)	0.0496 (3)	
O2	0.61027 (17)	0.55939 (15)	0.10512 (5)	0.0524 (3)	
O3	−0.20663 (14)	0.67321 (13)	−0.17341 (4)	0.0372 (3)	
O4	−0.43858 (14)	0.83254 (13)	−0.12867 (4)	0.0375 (3)	

### Atomic displacement parameters (Å^2^)

**Table:**

	*U*^11^	*U*^22^	*U*^33^	*U*^12^	*U*^13^	*U*^23^
C1	0.0407 (8)	0.0377 (7)	0.0246 (7)	−0.0091 (5)	0.0069 (6)	−0.0012 (5)
C2	0.0509 (9)	0.0481 (8)	0.0260 (7)	−0.0077 (7)	0.0000 (7)	0.0020 (6)
C3	0.0426 (9)	0.0416 (7)	0.0348 (8)	−0.0005 (6)	−0.0047 (7)	0.0043 (6)
C4	0.0348 (8)	0.0339 (7)	0.0344 (8)	−0.0014 (5)	0.0014 (6)	0.0030 (5)
C5	0.0301 (7)	0.0271 (6)	0.0263 (7)	−0.0030 (4)	0.0045 (5)	0.0022 (4)
C6	0.0276 (7)	0.0285 (6)	0.0280 (7)	−0.0012 (4)	0.0078 (5)	0.0011 (4)
C7	0.0279 (7)	0.0248 (6)	0.0237 (7)	−0.0032 (4)	0.0076 (5)	0.0012 (4)
C8	0.0283 (7)	0.0299 (6)	0.0248 (7)	−0.0030 (4)	0.0093 (5)	−0.0020 (4)
C9	0.0309 (7)	0.0327 (6)	0.0218 (6)	−0.0065 (5)	0.0073 (5)	−0.0014 (4)
C10	0.0243 (7)	0.0320 (6)	0.0283 (7)	−0.0032 (5)	0.0044 (5)	0.0026 (5)
C11	0.0276 (7)	0.0312 (6)	0.0274 (7)	−0.0003 (4)	0.0089 (5)	−0.0009 (5)
C12	0.0281 (7)	0.0265 (6)	0.0243 (6)	−0.0031 (4)	0.0073 (5)	0.0011 (4)
C13	0.0322 (7)	0.0305 (6)	0.0256 (7)	−0.0029 (5)	0.0094 (6)	−0.0017 (4)
C14	0.0333 (7)	0.0286 (6)	0.0240 (7)	−0.0065 (5)	0.0069 (5)	0.0005 (4)
C15	0.0452 (9)	0.0650 (9)	0.0254 (7)	−0.0003 (7)	0.0114 (6)	−0.0089 (6)
C16	0.0267 (7)	0.0530 (8)	0.0431 (9)	0.0020 (6)	0.0081 (6)	0.0042 (6)
O1	0.0507 (7)	0.0691 (7)	0.0311 (6)	−0.0053 (5)	0.0150 (5)	−0.0111 (5)
O2	0.0393 (7)	0.0647 (7)	0.0473 (7)	0.0151 (5)	0.0029 (5)	−0.0009 (5)
O3	0.0324 (6)	0.0543 (6)	0.0226 (5)	−0.0008 (4)	0.0041 (4)	−0.0040 (4)
O4	0.0275 (6)	0.0520 (6)	0.0292 (5)	0.0027 (4)	0.0021 (4)	0.0007 (4)

### Geometric parameters (Å, °)

**Table:**

C1—O1	1.2233 (18)	C9—O3	1.3570 (16)
C1—C14	1.4806 (19)	C9—C10	1.4356 (19)
C1—C2	1.482 (2)	C10—O4	1.3603 (16)
C2—C3	1.328 (2)	C10—C11	1.3637 (19)
C2—H2	0.94	C11—C12	1.4213 (19)
C3—C4	1.471 (2)	C11—H11	0.94
C3—H3	0.94	C12—C13	1.4116 (19)
C4—O2	1.2248 (18)	C13—C14	1.374 (2)
C4—C5	1.4856 (19)	C13—H13	0.94
C5—C6	1.3755 (19)	C15—O3	1.4273 (18)
C5—C14	1.4249 (19)	C15—H15A	0.97
C6—C7	1.4078 (18)	C15—H15B	0.97
C6—H6	0.94	C15—H15C	0.97
C7—C8	1.4165 (18)	C16—O4	1.4231 (18)
C7—C12	1.4262 (19)	C16—H16A	0.97
C8—C9	1.3659 (19)	C16—H16B	0.97
C8—H8	0.94	C16—H16C	0.97
			
O1—C1—C14	122.12 (14)	O4—C10—C9	113.79 (12)
O1—C1—C2	120.49 (13)	C11—C10—C9	120.42 (12)
C14—C1—C2	117.39 (13)	C10—C11—C12	120.58 (12)
C3—C2—C1	122.14 (14)	C10—C11—H11	119.7
C3—C2—H2	118.9	C12—C11—H11	119.7
C1—C2—H2	118.9	C13—C12—C11	122.39 (12)
C2—C3—C4	122.70 (14)	C13—C12—C7	118.71 (12)
C2—C3—H3	118.7	C11—C12—C7	118.90 (12)
C4—C3—H3	118.7	C14—C13—C12	121.39 (13)
O2—C4—C3	120.97 (13)	C14—C13—H13	119.3
O2—C4—C5	121.61 (13)	C12—C13—H13	119.3
C3—C4—C5	117.42 (13)	C13—C14—C5	119.72 (12)
C6—C5—C14	119.78 (12)	C13—C14—C1	120.17 (13)
C6—C5—C4	120.19 (13)	C5—C14—C1	120.11 (12)
C14—C5—C4	120.03 (12)	O3—C15—H15A	109.5
C5—C6—C7	121.23 (12)	O3—C15—H15B	109.5
C5—C6—H6	119.4	H15A—C15—H15B	109.5
C7—C6—H6	119.4	O3—C15—H15C	109.5
C6—C7—C8	121.38 (12)	H15A—C15—H15C	109.5
C6—C7—C12	119.13 (11)	H15B—C15—H15C	109.5
C8—C7—C12	119.48 (12)	O4—C16—H16A	109.5
C9—C8—C7	120.57 (12)	O4—C16—H16B	109.5
C9—C8—H8	119.7	H16A—C16—H16B	109.5
C7—C8—H8	119.7	O4—C16—H16C	109.5
O3—C9—C8	125.28 (12)	H16A—C16—H16C	109.5
O3—C9—C10	114.65 (12)	H16B—C16—H16C	109.5
C8—C9—C10	120.05 (12)	C9—O3—C15	116.71 (11)
O4—C10—C11	125.79 (12)	C10—O4—C16	116.88 (11)
			
O1—C1—C2—C3	−177.80 (14)	C10—C11—C12—C13	178.54 (11)
C14—C1—C2—C3	2.5 (2)	C10—C11—C12—C7	−0.80 (18)
C1—C2—C3—C4	1.9 (2)	C6—C7—C12—C13	1.57 (17)
C2—C3—C4—O2	175.88 (15)	C8—C7—C12—C13	−179.27 (10)
C2—C3—C4—C5	−4.0 (2)	C6—C7—C12—C11	−179.07 (10)
O2—C4—C5—C6	2.1 (2)	C8—C7—C12—C11	0.09 (17)
C3—C4—C5—C6	−178.01 (11)	C11—C12—C13—C14	−179.95 (10)
O2—C4—C5—C14	−178.31 (13)	C7—C12—C13—C14	−0.61 (19)
C3—C4—C5—C14	1.54 (18)	C12—C13—C14—C5	−1.28 (19)
C14—C5—C6—C7	−1.29 (18)	C12—C13—C14—C1	178.67 (10)
C4—C5—C6—C7	178.27 (10)	C6—C5—C14—C13	2.25 (18)
C5—C6—C7—C8	−179.76 (10)	C4—C5—C14—C13	−177.31 (11)
C5—C6—C7—C12	−0.62 (18)	C6—C5—C14—C1	−177.71 (11)
C6—C7—C8—C9	179.20 (10)	C4—C5—C14—C1	2.73 (17)
C12—C7—C8—C9	0.06 (18)	O1—C1—C14—C13	−4.4 (2)
C7—C8—C9—O3	−177.93 (10)	C2—C1—C14—C13	175.27 (11)
C7—C8—C9—C10	0.46 (19)	O1—C1—C14—C5	175.55 (12)
O3—C9—C10—O4	−3.06 (16)	C2—C1—C14—C5	−4.77 (18)
C8—C9—C10—O4	178.39 (11)	C8—C9—O3—C15	−10.22 (19)
O3—C9—C10—C11	177.38 (11)	C10—C9—O3—C15	171.32 (11)
C8—C9—C10—C11	−1.16 (19)	C11—C10—O4—C16	−6.25 (19)
O4—C10—C11—C12	−178.17 (10)	C9—C10—O4—C16	174.23 (11)
C9—C10—C11—C12	1.33 (19)		
